# Artificial Intelligence Competencies and Educational Needs Among ERNICA Members: Results of a Multinational Survey

**DOI:** 10.1055/a-2787-2213

**Published:** 2026-02-03

**Authors:** Holger Till, Hesham Elsayed, Beate Obermüller, Richard Gnatzy, Martin Lacher, Sebastian Tschauner, Rosa Verhoeven, Rene M. H. Wijnen, Georg Singer, ERNICA AI Task Force Members, Sara Roman Galdran, Lucas Matthijssens, Roel Bakx, Francesca Russo, Jan Hulscher, Michael Aertsen, Leopoldo Martínez, Michael Boettcher, Federico Scorletti, Frédéric Hameury, Richard Wagner, Miriam Duci

**Affiliations:** 1 Department of Pediatric and Adolescent Surgery, Medical University of Graz, Graz, Austria; 2 Department of Pediatric Surgery, University of Leipzig, Leipzig, Germany; 3 Division of Pediatric Radiology, Department of Radiology, Medical University of Graz, Graz, Austria; 4 Division of Pediatric Surgery, Department of Surgery, University Medical Center Groningen, Groningen, The Netherlands; 5 Department of Pediatric Surgery, Erasmus MC Sophia Children's Hospital, Rotterdam, The Netherlands; Department of Pediatric Surgery, Erasmus Medical Center, The Netherlands; Department of Pediatric Surgery, University Hospital Ghent (UZ Gent), Belgium; Department of Pediatric Surgery, Amsterdam University Medical Center, The Netherlands; Department of Pediatric Surgery, University Hospital Leuven (UZ Leuven), Belgium; Department of Pediatric Surgery, University Medical Center Groningen, The Netherlands; Department of Pediatric Surgery, University Hospital Leuven (UZ Leuven), Belgium; Department of Pediatric Surgery, Hospital Universitario La Paz, Madrid, Spain; Department of Pediatric Surgery, University Hospital Heidelberg, Germany; Department of Pediatric Surgery, Ospedale Pediatrico Bambino Gesù, Rome, Italy; Department of Pediatric Surgery, CHU de Lyon, France; Department of Pediatric Surgery, University Hospital Leipzig, Germany; Department of Pediatric Surgery, University of Padova, Italy

**Keywords:** artificial intelligence (AI), ERNICA, survey, educational needs, competencies

## Abstract

**Introduction:**

Artificial intelligence (AI) is increasingly recognized as a transformative force in healthcare. In the field of rare diseases, AI can enhance diagnostic accuracy and facilitate knowledge-sharing across borders. To effectively contribute to the development and use of AI-based medical support systems, clinicians must provide specialized AI competencies. This survey assesses the AI readiness, educational needs, and perceptions of members within the European Reference Network for Rare Inherited and Congenital Anomalies (ERNICA).

**Methods:**

A structured online survey consisting of 22 questions was distributed to 389 ERNICA members, collecting data on demographics, AI awareness, current use, educational needs, concerns, and future expectations.

**Results:**

A total of 89 members responded (23%), representing a multidisciplinary group with varying experience. Most respondents (94%) reported no formal AI training yet, and rated their AI knowledge as basic (66%) or intermediate (26%). About 48% of the participants stated using AI applications already. Key educational needs included online courses and webinars. Major concerns focused on the reliability and accuracy of AI tools (80%) and ethical implications (71%). At the same time, 55% expect ERNICA to take a leading role in AI education in the diagnosis and management of rare gastrointestinal diseases.

**Conclusion:**

This survey among ERNICA members revealed a definite gap of AI understanding and training. Addressing these issues requires tailored educational initiatives focused on practical AI applications, ethical considerations, and interpretability. By adopting a proactive role in AI capacity-building, ERNICA could contribute to responsible and effective integration of AI into rare disease care.

## Introduction

Although artificial intelligence (AI) is increasingly applied across healthcare, its role in supporting the diagnosis and management of rare congenital anomalies—central to the mission of the European Reference Network for rare Inherited and Congenital Anomalies (ERNICA)—remains underexplored and urgently requires clarification. 1 2 3 4 In rare diseases, where diagnostic complexity, fragmented care pathways, and limited data pose persistent challenges, AI may offer substantial support in improving diagnosis, risk stratification, and clinical decision-making to potentially anybody worldwide at any time. 5 6 7

Despite the rising prominence of AI in healthcare, there is still a substantial gap between awareness and practical AI competencies of healthcare professionals. 8 For many clinicians, AI remains a largely non-transparent concept—frequently referenced but poorly understood. This knowledge gap could be especially significant in multidisciplinary teams where diverse roles and skill sets influence how AI is perceived and used. 9

ERNICA brings together pediatric surgeons, pediatricians, radiologists, geneticists, nurses, and patient representatives across Europe and beyond. Its transnational and multidisciplinary structure makes it both a unique model of collaborative care and a challenging setting to explore how emerging technologies like AI are integrated into practice. Through initiatives such as workshops, conferences, webinars, and collaborative projects, ERNICA could serve as a catalyst for the integration of AI in pediatric surgery.

Understanding the current level of AI awareness, application, and educational need among ERNICA members is crucial to fostering safe, effective, and equitable AI adoption. This study therefore aimed to assess these factors via a structured survey. We hypothesized that although ERNICA members are aware of AI and its potential, they would report limited practical knowledge and express a strong demand for structured education and guidance on its clinical application in rare congenital anomalies.

## Methods

### Study Design and Population

This was a cross-sectional descriptive study conducted via an online survey. The target population included all registered ERNICA members.

### Survey Development

The survey was developed by a multidisciplinary working group consisting of pediatric surgeons, medical educators, and researchers with experience in AI applications within the AI task force of ERNICA, drawing on existing literature about AI competency surveys in healthcare 8 and tailored to the context of rare diseases ( Supplementary Table S1 , available in online version only). Both closed-ended and open-ended questions were included to capture quantitative data and qualitative perspectives. The questions were grouped into the following five main sections:

1. Demographics and professional background
2. AI awareness and self-assessed understanding
3. Current AI usage and application areas
4. Educational needs and preferred learning formats
5. Perceived barriers, ethical concerns, and future perspectives

To ensure content validity, the draft survey was reviewed by the AI task force members of ERNICA, who assessed item clarity, relevance, and completeness. Their feedback led to minor revisions in wording and structure. The revised survey was then pilot tested internally to assess comprehensibility and flow. Based on the pilot feedback, minor adjustments were made to the phrasing of open-ended items and the order of selected questions.

### Survey Distribution

The survey was disseminated via email to all 389 ERNICA members using the official ERNICA contact database. It was conducted anonymously using SurveyMonkey as a secure online survey platform. The invitation included a brief explanation of the survey's purpose and an estimate of completion time (approximately 15 minutes). The survey remained open for responses for 4 weeks. Two reminders were sent during that period.

### Data Handling and Analysis

Responses were exported to Microsoft Excel and analyzed using descriptive statistics. Quantitative data such as age range and AI usage frequency were summarized using absolute numbers and percentages. Qualitative responses (such as open-ended answers regarding AI concerns or desired educational content) were grouped in thematic patterns but not formally coded; the most common answers are displayed. Categorical data comparisons were performed with the chi-squared test. A *p* -value < 0.05 was considered statistically significant.

### Ethical Considerations

Participation in the survey was voluntary and anonymous. No identifiable personal data were collected. The ethical committee of the Medical University of Graz approved the study (protocol code 1202/2024).

## Results

### Demographics and Professional Background

A total of 89 members participated in the survey, corresponding to a response rate of 22.9%. While the majority of the respondents were over 50 years old ( *n* = 38, 43%), *n* = 31 (35%) were 41 to 50 years, *n* = 19 (21%) were 30 to 40 years, and *n* = 1 (1%) <30 years old. In terms of professional experience 35 participants (39%) had 11 to 20 years of experience, *n* = 33 (37%) had more than 20 years, *n* = 16 (18%) had 5 to 10 years, and *n* = 5 (6%) had less than 5 years. The vast majority ( *n* = 80, 89%) stated to work in academic medical centers, only *n* = 4 (5%) worked in community hospitals and *n* = 5 (6%) in other institutions. More than half of the participants were pediatric surgeons ( *n* = 49, 55%), *n* = 10 (11%) were pediatric gastroenterologists, *n* = 9 (10%) were neonatologists, *n* = 4 (5%) were patient representatives, and *n* = 16 (18%) others including for instance radiologists, anesthesiologists, dieticians, and nurse practitioners.

### AI Awareness and Self-assessed Understanding

**Fig. 1:**
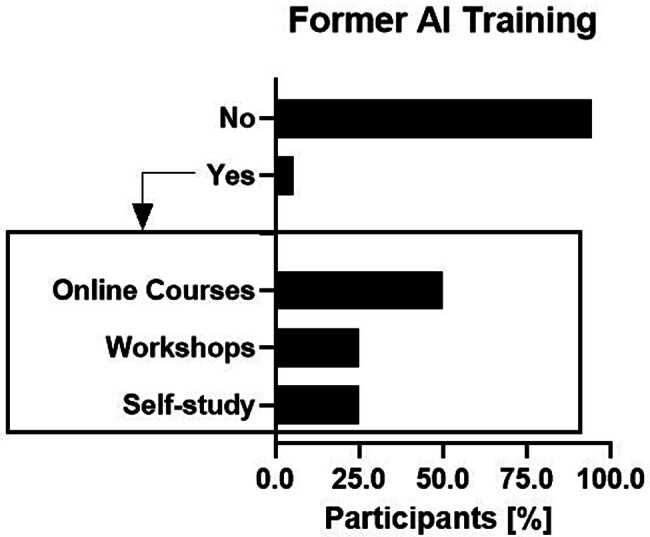
Former AI training the respondents have received.

Most respondents rated their general understanding of AI as either basic ( *n* = 59, 66%) or intermediate ( *n* = 23, 26%); *n* = 7 (8%) stated that they have no general understanding. None of the participants rated their understanding as advanced. Only a minority ( *n* = 5, 6%) reported having received former AI training, which included online courses, workshops, and self-study ( Fig. 1 ).

**Fig. 2:**
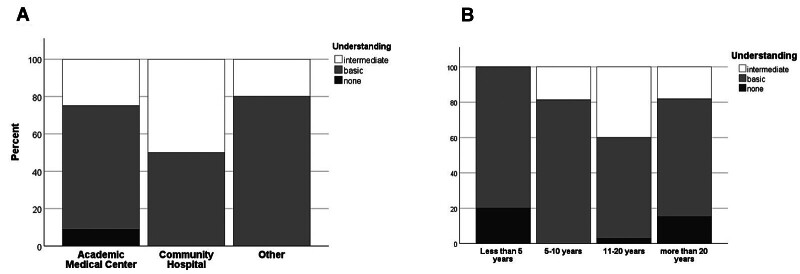
Comparisons of the self-assessed understanding of AI regarding workplace ( **A** ) and years of professional experience ( **B** ).

The self-assessed understanding of AI was not statistically different in the various institutions the participants work in (χ ^2^ = 2.081, *p* = 0.721). The years of professional experience, however, almost reached statistical significance (χ ^2^ = 11.730, *p* = 0.068). These findings are depicted in Fig. 2 .

**Fig. 3:**
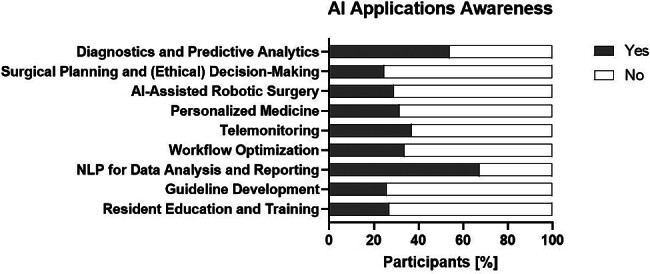
AI applications the respondents are aware of.

When asked about specific AI applications they are aware of, natural language processing (NLP) for data analysis and reporting were most commonly mentioned (67%), followed by diagnostics and predictive analytics (54%). The least awareness was reported with guideline development (26%), surgical planning and (ethical) decision-making (25%). Detailed findings are depicted in Fig. 3 .

### Current AI Usage and Application Areas

**Fig. 4:**
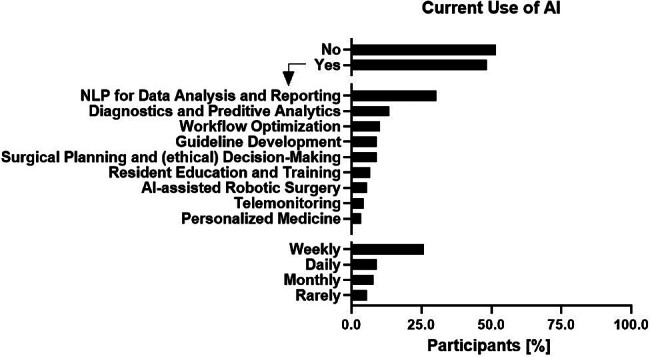
Current use of AI in professional healthcare among participants.

While almost half of the participants stated that they use AI applications ( *n* = 43, 48%), *n* = 46 (52%) stated that they do not use AI in their practice. The most frequently cited AI applications being used were NLP for data analysis and reporting (30%), diagnostics and predictive analytics (14%), and workflow optimization (10%). Most respondents stated that they use AI applications on weekly basis ( *n* = 23, 26%) ( Fig. 4 ).

When asked about their primary needs for AI tools, respondents identified NLP for data analysis and reporting (72%) and diagnostics and predictive analytics (61%) as the top priorities. Details are shown in Supplementary Fig. S1 (available in online version only).

### Educational Needs and Training Tailored for ERNICA

**Fig. 5:**
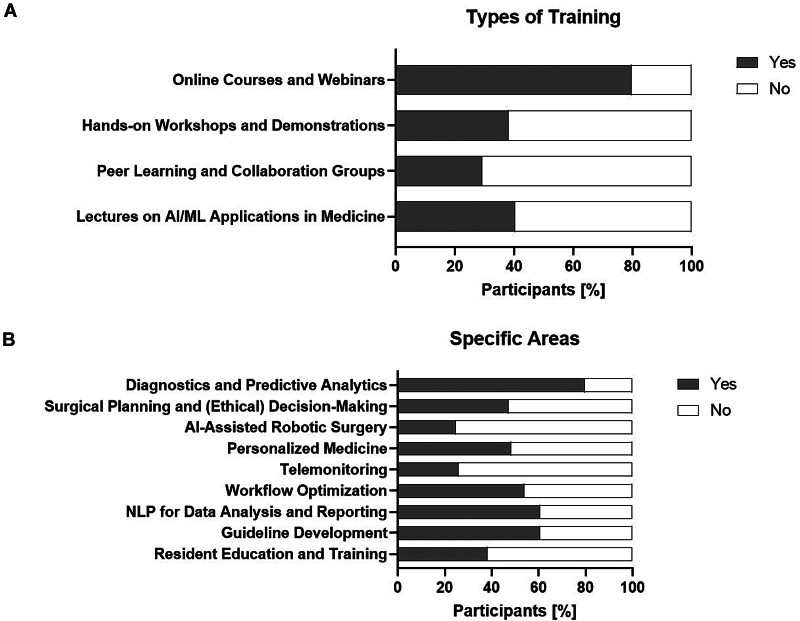
Preferred learning format ( **A** ), and top priority areas ( **B** ).

The vast majority of respondents ( *n* = 81, 91%) expressed a general interest for AI training specifically adapted to the ERNICA context. About 80% of the participants considered online courses and webinars as the preferred learning format. The top three priority areas for AI education were diagnostics and predictive analytics (80%), guideline development (61%) and NLP for data analysis and reporting (61%). Detailed results are presented in Fig. 5 .

### Perceived Barriers, Ethical Concerns, and Future Perspectives

When asked about barriers to AI adoption, *n* = 61 (69%) of the respondents expressed some concerns, *n* = 13 (15%) significant concerns, and *n* = 7 (8%) no concerns, while *n* = 8 (9%) were unsure. Neither age group nor years of experience had a significant influence on these concerns (χ ^2^ = 3.879, *p* = 0.919 and χ ^2^ = 4.795, *p* = 0.852, respectively).

The main concerns were reliability and accuracy of AI tools (80%) and ethical issues (71%) followed by loss of surgical skills (38%), patient's acceptance and trust (27%), and cost and resource allocation (25%).

All but one participant ( *n* = 1, 1%) stated to have concerns regarding data bias in AI algorithms affecting healthcare outcomes (extremely concerned *n* = 12, 14%; very concerned *n* = 21, 24%; moderately concerned *n* = 44, 49%; slightly concerned *n* = 11, 12%).

Perceived causes of bias in AI algorithms included bias in data collection processes (81%), misinterpretation of data by algorithms (71%), poor data quality or preprocessing (64%), and lack of diversity in training data (62%).

Nevertheless, a majority ( *n* = 49, 55%) anticipated AI tools becoming integral to daily practice, *n* = 35 (39%) anticipated AI to be limited to specific applications, *n* = 4 (5%) were unsure, and one participant (1%) expected AI to have minimal impact.

Anticipated benefits were better patient outcomes (83%), improved diagnostic accuracy (83.1%), cost savings (39.3%), enhanced surgical precision (33%), and reduced operation times (20%).

The question “Which AI applications or advancements would you like to see implemented in ERNICA?” was answered with “AI-based diagnostic and/or prognostic algorithms” 25 times and guideline development and research 8 times each. However, “do not know,” “too limited background,” and “not sure” and no answer were given 42 times.

The question “Are there any existing AI tools or applications that you find particularly useful for ERNICA?” was answered with “AI algorithms for preoperative image analysis” seven times, “chat bots” seven times, and “diagnostic tools” six times. “Do not know,” “too limited background,” “unsure,” and no answer was given 55 times.

More than half of the participants expect organizations like ERNICA to take a leading role in AI education in the diagnosis and management of rare digestive/diaphragmatic/abdominal wall diseases and anomalies ( *n* = 49, 55%); *n* = 34 (38%) were unsure and *n* = 6 (7%) answered this question with no.

## Discussion

### Demographics and Professional Background

This survey provides valuable insights into AI readiness within the ERNICA network. Respondents represented a multidisciplinary spectrum—including pediatric surgeons, gastroenterologists, neonatologists, and other rare disease specialists—reflecting the collaborative nature of the organization. The moderate response rate (23%) suggests that engagement with AI remains largely confined to clinicians already interested in digital innovations. 9 10 11 Although the diversity of professional backgrounds is a strength, it may also contribute to variation in AI familiarity, highlighting the need for customized educational programs to close knowledge gaps and promote shared AI competencies across the network.

### AI Awareness and Self-assessed Understanding

General awareness of AI's potential was widespread among ERNICA members, yet actual understanding and confidence remain limited. Most respondents rated their AI knowledge as basic, with only a minority reporting formal training or advanced familiarity. Notably, neither institutional type nor years of professional experience appeared to influence self-assessed AI understanding. This gap between perceived potential and practical competence mirrors observations in other pediatric surgical communities, such as in a recent ESPES (European Society of Paediatric Endoscopic Surgeons) survey. 8 Nevertheless, over half of the participants were aware of NLP for data analysis and reporting as well as diagnostics and predictive analytics.

Clinicians recognize AI's potential to improve diagnostics, 12 imaging, 13 and personalized treatment planning, but many feel unequipped to interpret AI outputs or participate in AI-driven projects. Bridging this gap through foundational AI literacy is essential for safe and effective AI integration, particularly in rare disease care, where decisions are complex and evidence is often limited.

### Current AI Usage and Application Areas

Despite limited formal training, some ERNICA members have begun integrating AI into clinical workflows, primarily in imaging, diagnostics, and predictive analytics. Broader implementation, including surgical planning and decision-support systems, especially in rare diseases remains modest. 14 15 16 17 18 19 Key barriers identified include limited access to validated AI tools, lack of institutional infrastructure, and perceptions that existing solutions do not fit the workflows of rare disease care. 11 20 Without addressing these hurdles, AI's full potential to improve rare disease outcomes may remain unrealized within ERNICA.

### Educational Needs and Preferred Learning Formats

The survey revealed a strong demand for AI education, with respondents favoring clinically relevant and flexible formats such as hands-on workshops, modular online courses, and case-based webinars. 21 22 There was also a call for role-specific educational content tailored to pediatric surgeons, gastroenterologists, geneticists, and other specialists. Collaboration between clinicians and data scientists could enhance AI tool design, build trust, and foster mutual learning. As a transnational, multidisciplinary network, ERNICA is well-positioned to lead these educational efforts by developing shared resources, mentorship structures, and core competencies for AI literacy tailored to rare disease care.

### Perceived Barriers, Ethical Concerns, and Future Perspectives

Beyond knowledge gaps, the survey highlights significant barriers and ethical concerns that may hinder responsible AI adoption including algorithmic bias, data privacy, and the opacity of AI systems—issues particularly relevant in rare diseases due to small datasets and patient variability. 23 Explainable AI (XAI), which makes AI decision-making transparent and interpretable, was emphasized as essential. 24 Practical obstacles such as limited institutional support, restricted access to AI solutions, and workflow mismatches—referring to misalignments between how AI tools function and how clinical tasks are actually performed—further impede adoption.

Overcoming these barriers by cloud solutions and freeware may foster institutional deployment. 25 Cloud-based solutions and open-access platforms may help overcome these challenges, enabling pilot projects, centralized procurement of validated tools, and cross-border data-sharing. ERNICA could also advocate for governance frameworks, infrastructure investment, and co-creation of AI tools with technology developers to ensure ethical, reliable, and context-appropriate solutions. Its pan-European scope and multidisciplinary mandate position ERNICA to champion ethical standards, foster clinician–AI collaboration, and drive the digital transformation of rare disease care. Challenges such as limited funding, heterogeneity of member engagement, and regulatory barriers must be considered to ensure sustainable integration.

### Limitations

This study has several limitations that should be considered when interpreting the findings. First, the response rate was relatively low, with only 89 out of 389 ERNICA members participating, which may limit the representativeness and generalizability of the results. However, this selection bias may also suggest that general awareness and understanding could even be lower in a broader population underscoring the need for education and training in AI. The respondent pool was predominantly composed of individuals from academic institutions, potentially underrepresenting the perspectives of clinicians working in community hospitals or private practices. Nevertheless, the cohort was multidisciplinary and the survey is the first AI survey among ERNICA members. Moreover, the nature of the questions has only allowed descriptive statistics, and subgroup analysis was confined to a comparison of the self-assessed understanding of AI concerning the various institutions the participants work in and the years of professional experience of the participants. Additionally, the reliance on self-reported data introduces the possibility of recall bias and subjective inaccuracies in evaluating AI knowledge and usage. Additionally, utilizing an objective AI competency test may overcome the self-report bias. However, such tools still have to be developed. The survey did not assess the long-term retention of AI-related knowledge or its practical application in clinical settings, limiting insights into real-world impact. Lastly, the geographic concentration of respondents may constrain the extrapolation of findings to a global context, particularly regarding international trends in AI adoption.

## Conclusion

A foundational knowledge of AI is critical to empower clinicians to contribute meaningfully to the development and refinement of AI tools. The findings from this survey provide a foundation for the development of AI competency-building within the ERNICA network. The development of AI and ML algorithms heavily relies on large datasets. In pediatric surgery, however, many diseases and malformations are rare, which limits the availability of such extensive data. Multinational, interdisciplinary networks (such as ERNICA) could therefore play an important role in overcoming this challenge. By offering targeted educational initiatives, fostering interdisciplinary collaborations, and addressing both ethical concerns and infrastructural barriers, ERNICA could play a role in integrating AI into rare disease care, paving the way for its safe and responsible adoption.

Future research should build on these findings by examining the long-term effects of AI education in clinical settings, assessing hands-on trainings and developing strategies to address ethical issues and algorithmic bias, supporting the responsible integration of AI into rare disease care. Moreover, multicentric AI pilot projects and collaborations with data scientists could contribute to a broader understanding of AI within the ERNICA network. In this context, ERNICA could play a role in developing a dedicated AI core curriculum and organizing annual training programs.
