# Supplementary material for: Artificial Intelligence Competencies and Educational Needs Among ERNICA Members: Results of a Multinational Survey
Source: Eur J Pediatr Surg. 2026 Feb 3;36(4):324–9. doi: 10.1055/a-2787-2213 (PMC13375283; doi:10.1055/a-2787-2213)
Supplement: Supplementary file 1 — Supplementary Material [file 10-1055-a-2787-2213_29127028.pdf]

ERNICA AI TASK FORCE MEMBERS:

- Sara Roman Galdran, Department of Pediatric Surgery, Erasmus Medical Center, The Netherlands
- Lucas Matthijssens, Department of Pediatric Surgery, University Hospital Ghent (UZ Gent), Belgium
- Roel Bakx, Department of Pediatric Surgery, Amsterdam University Medical Center, The Netherlands
- Francesca Russo, Department of Pediatric Surgery, University Hospital Leuven (UZ Leuven), Belgium
- Jan Hulscher, Department of Pediatric Surgery, University Medical Center Groningen, The Netherlands
- Michael Aertsen, Department of Pediatric Surgery, University Hospital Leuven (UZ Leuven), Belgium
- Leopoldo Martínez, Department of Pediatric Surgery, Hospital Universitario La Paz, Madrid, Spain
- Michael Boettcher, Department of Pediatric Surgery, University Hospital Heidelberg, Germany
- Federico Scorletti, Department of Pediatric Surgery, Ospedale Pediatrico Bambino Gesù, Rome, Italy
- Frédéric Hameury, Department of Pediatric Surgery, CHU de Lyon, France
- Richard Wagner, Department of Pediatric Surgery, University Hospital Leipzig, Germany
- Miriam Duci, Department of Pediatric Surgery, University of Padova, Italy

Supplementary Figure 1. Primary needs for AI tools

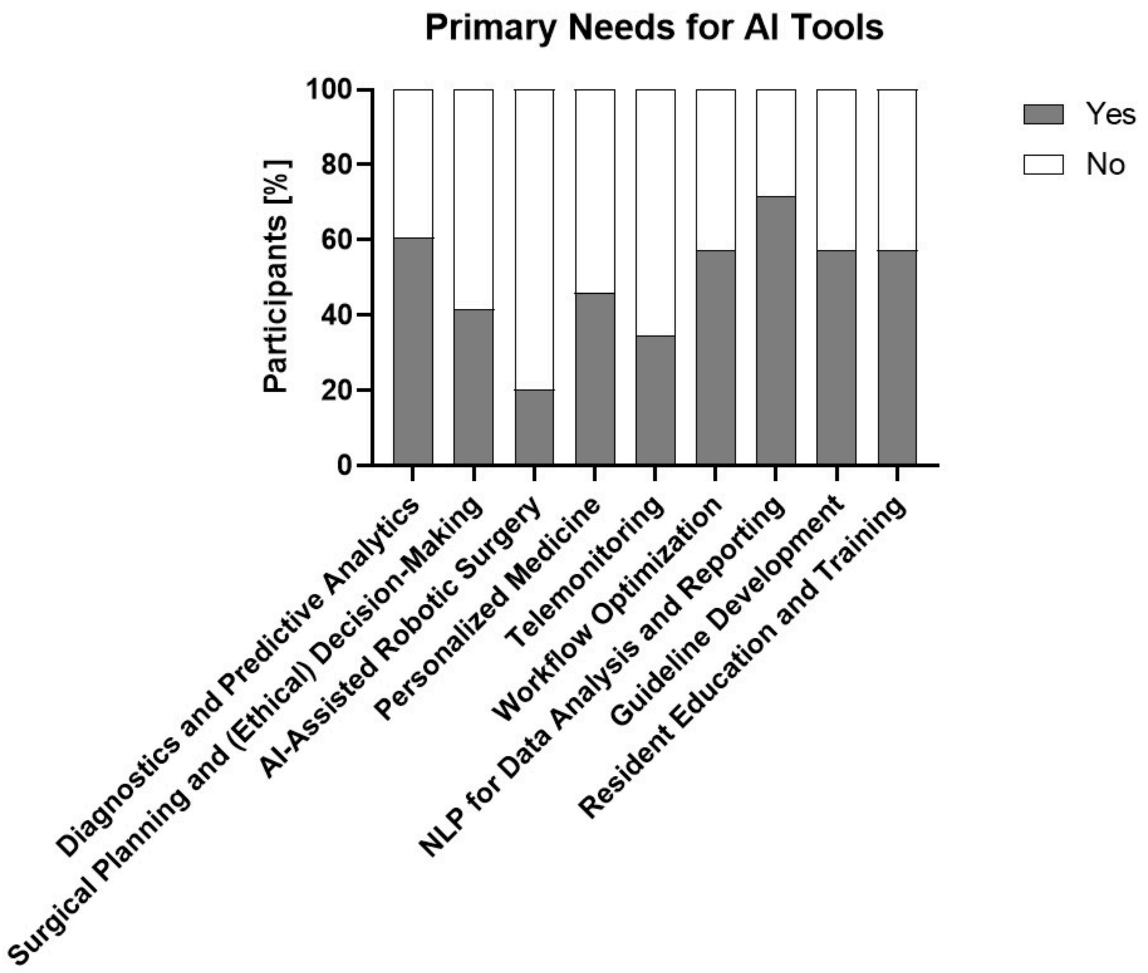

Supplementary Table 1: Survey “Artificial Intelligence Competencies and Educational Needs Among ERNICA Members: Results of a Multinational Survey”

***Section 1:** Demographics and Professional Background*

1. What is your age range? (single choice)	• <30 years
	• 30-40 years
	• 41-50 years
	• >50 years

2. Years of medical experience in your current role (single choice)	• Less than 5 years
	• 5-10 years
	• 11-20 years
	• >20 years

3. What type of institution do you primarily work in? (single choice)	• Academic Medical Center
	• Community Hospital
	• Private Pratice
	• Other

4. What is your professional background? (single choice)	• Pediatric Surgeon
	• Neonatologist
	• Pediatric Gastroenterologist
	• Pediatric Pulmonologist
	• Clinical Geneticist
	• Nurse
	• Specialized Nurse/Nurse Practitioner
	• Patient Representative
	• Parent
	• Patient
	• Other (please specify)

Section 2: *AI Awareness and Self-Assessed Understanding*

5. How would you rate your general understanding of AI? (single choice)	• none
	• basic
	• intermediate
	• advanced

6. Have you received any formal training in Artificial Intelligence (AI)?	• yes
	○ online courses
	○ workshops
	○ Formal Academic Studies
	○ Self studies
	○ Other (please specify)
	• no

7. Which of the following AI applications are you aware of in pediatric surgery? (Check all that apply)	• Diagnostics and Predictive Analytics
	• Surgical Planning and (Ethical) Decision-Making
	• AI-Assisted Robotic Surgery
	• Personalized Medicine
	• Telemonitoring
	• Workflow Optimization
	• NLP für Data Analysis and Reporting
	• Guideline Development
	• Resident Education and Training

Section 3: *Current AI Usage and Application Areas*

8. Do you currently use any AI tools in your practice?	• yes
	○ Diagnostics and Predictive Analytics
	○ Surgical Planning and (Ethical) Decision-Making
	○ AI-Assisted Robotic Surgery
	○ Personalized Medicine
	○ Telemonitoring
	○ Workflow Optimization

	○ NLP für Data Analysis and Reporting
	○ Guideline Development
	○ Resident Education and Training
	• no

9. How frequently do you use AI tools in your practice? (single choice)	• Daily
	• Weekly
	• Monthly
	• Rarely

10. How would you describe your primary needs for AI tools? (Select all that apply)	• Diagnostics and Predictive Analytics
	• Surgical Planning and (Ethical) Decision-Making
	• AI-Assisted Robotic Surgery
	• Personalized Medicine
	• Telemonitoring
	• Workflow Optimization
	• NLP für Data Analysis and Reporting
	• Guideline Development
	• Resident Education and Training

Section 4: *Educational Needs and Preferred Learning Formats*

11. Would you be interested in receiving more training in AI specifically tailored for the diagnosis and management of rare digestive/diaphragmatic/abdominal wall diseases and anomalies? (single choice)	• yes
	• no

12. What types of training or educational resources would be most helpful?	• Online Courses and Webinars
	• Hands-on Workshops and Demonstrations
	• Peer Learning and Collaboration Groups
	• Lectures on AI/ML Applications in Medicine

13. Which specific areas of AI would you like to learn more about? (Select all that apply)	• Diagnostics and Predictive Analytics
	• Surgical Planning and (Ethical) Decision-Making
	• AI-Assisted Robotic Surgery
	• Personalized Medicine
	• Telemonitoring
	• Workflow Optimization
	• NLP für Data Analysis and Reporting
	• Guideline Development
	• Resident Education and Training

Section 5: *Perceived Barriers, Ethical Concerns and Future Perspectives*

14. Do you have general concerns about implementing AI technologies in your practice? (single choice)	• Yes, significant concerns
	• Yes, some concerns
	• No concerns
	• unsure

15. What are your main concerns regarding AI in pediatric surgery? (check all that apply)	• Ethical issues (e.g., Bias, Privacy)
	• Reliability and Accuracy of AI Tools
	• Loss of Surgical Skills
	• Cost and Resource Allocation
	• Patient Acceptance and Trust

16. How concerned are you about data bias in AI algorithms affecting healthcare outcomes? (single choice)	• Extremely concerned
	• Very concerned
	• Moderately concerned
	• Slightly concerned
	• Not concerned

17. What do you believe are the primary causes of data bias in AI algorithms? (check all that apply)	• Lack of diversity in training data
	• Poor data quality and preprocessing
	• Bias in data collection processes
	• Misinterpretation of data by algorithms
	• All of the above

18. How do you see the role of AI evolving in pediatric surgery over the next 10 years?	• Integral to daily practice
	• Limited to specific applications
	• Minimal impact
	• Uncertain

19. What do you see as the primary benefits of integrating AI into pediatric surgery? (check all that apply)	• Improved diagnostic accuracy
	• Enhanced surgical precision
	• Reduced operation times
	• Cost savings
	• Better patient outcomes
	• Other (please specify)

20. Which AI applications or advancements would you like to see implemented in ERNICA? (open-ended)	• open ended answer

21. Are there any existing AI tools or applications that you find particularly useful for ERNICA? (open-ended)	• open ended answer

22. Do you expect organizations like ERNICA to take a leading role in AI education in the diagnosis and management of rare digestive/diaphragmatic/abdominal wall diseases and anomalies? (single choice)	• yes
	• no
	• not sure
